# Redox-driven mitochondrial DNA stress in hepatocellular carcinoma: innate immune remodelling, tumour immune escape, and immunotherapy implications

**DOI:** 10.3389/fimmu.2026.1903643

**Published:** 2026-09-04

**Authors:** Ze Chang, Guannan Liu, Jinshan Zhang, Hailin Lei, Haiyan Quan, Ying Piao

**Affiliations:** 1 Central Laboratory, The Affiliated Hospital of Yanbian University, Yanji, China; 2 Emergency Department, The Affiliated Hospital of Yanbian University, Yanji, China; 3 Department of Metabolic and Bariatric Surgery, The First Affiliated Hospital of Jinan University, Guangzhou, China

**Keywords:** hepatocellular carcinoma, immunotherapy, innate immunity, mitochondrial danger signals, mitochondrial DNA, redox signalling, tumour immune escape, tumour immune microenvironment

## Abstract

Hepatocellular carcinoma (HCC) develops in a chronically injured liver where metabolic adaptation, oxidative stress, innate immune signalling, and immune tolerance are already intertwined. Mitochondria connect these processes: they sustain tumour-cell fitness, yet damaged organelles expose mitochondrial DNA (mtDNA) as an intracellular and intercellular danger signal. Persistent reactive oxygen species, altered mitochondrial dynamics, nucleoid instability, and incomplete mitophagy–lysosomal clearance can oxidise, fragment, and displace mtDNA. The resulting material may remain in the cytosol, circulate freely or in protein-associated complexes, or be transferred within extracellular vesicles. These forms are not immunologically equivalent. Cytosolic mtDNA favours cGAS–STING access; endocytosed material can engage endolysosomal TLR9; and oxidised mtDNA can cooperate with mitochondrial reactive oxygen species, ATP, cardiolipin, and ionic perturbation in NLRP3 inflammasome-associated signalling. Redox remodelling also alters interferon responsiveness, inflammasome competence, and myeloid-cell metabolism, allowing recipient cells to assign different meanings to a similar mitochondrial signal. We integrate these mechanisms into an acute immune activation–chronic immune adaptation continuum. Transient, spatially restricted, and efficiently cleared danger can support antigen presentation and effector recruitment, whereas recurrent or poorly cleared signalling can become embedded in suppressive myeloid remodelling, lymphocyte dysfunction, and spatial immune escape. Direct HCC studies support treatment-induced mtDNA–STING activation, hypoxic extracellular-vesicle-mediated mtDNA transfer, macrophage TLR9 signalling, and TFAM–mtDNA–NLRP3 coupling. The transition between immune states remains a testable synthesis, not an established linear pathway. Therapeutic intervention should be matched to signal form, recipient-cell competence, timing, spatial context, and hepatic reserve; pathway activation alone is an inadequate guide.

## 1 Introduction

Hepatocellular carcinoma (HCC), which accounts for most primary liver cancers, remains a major cause of cancer-related mortality despite advances in surveillance, locoregional therapy, molecularly targeted treatment, and immunotherapy (1, 2). Its aetiological distribution is changing: chronic hepatitis B virus (HBV) infection remains dominant in many regions, hepatitis C virus (HCV) infection and alcohol-related disease continue to contribute substantially, and metabolic dysfunction-associated steatotic liver disease (MASLD) and metabolic dysfunction-associated steatohepatitis (MASH) (3) account for a growing proportion of cases (4, 5). These conditions differ biologically, but all place malignant transformation within a liver already shaped by repeated injury, fibrosis, vascular remodelling, and physiological immune tolerance (1, 6, 7).

Systemic immunotherapy has shown that antitumour immunity can be restored in a subset of patients. Atezolizumab plus bevacizumab and tremelimumab plus durvalumab improved outcomes in advanced HCC (8, 9). By contrast, LEAP-002 did not meet its prespecified overall- and progression-free-survival thresholds (10), illustrating that adding checkpoint blockade to an active targeted backbone does not uniformly overcome resistance. Clinical benefit remains heterogeneous. The presence of an immune target does not identify the limiting immune defect. Tumours differ in aetiology, vascularisation, metabolic state, myeloid composition, antigen-presenting-cell competence, and the extent to which chronic liver disease has already remodelled innate and adaptive immunity (6, 7). A mechanistic account of treatment response must explain how tumour-cell injury is communicated to surrounding cells and why an inflammatory signal produces coordinated tumour control in one setting but chronic, tumour-supporting adaptation in another.

Mitochondria provide a plausible interface between tumour adaptation and immune communication. HCC cells reconfigure glucose, lipid, and amino-acid metabolism to survive hypoxia, nutrient limitation, and therapeutic pressure while retaining mitochondrial functions required for ATP production, biosynthesis, and redox buffering (11–14). This compensation can preserve cell viability without fully restoring mitochondrial membranes, respiratory function, nucleoid organisation, or quality control. A normally sequestered mitochondrial genome can consequently become oxidised, fragmented, displaced into the cytosol, or released between cells, converting metabolic stress into an intracellular and intercellular danger signal (15, 16).

The immune meaning of mtDNA is determined both before and after receptor engagement. Cytosolic mtDNA can activate cyclic GMP–AMP synthase (cGAS)–stimulator of interferon genes (STING) (15, 16); endocytosed mtDNA can reach Toll-like receptor 9 (TLR9) (16, 17); and oxidised mtDNA can contribute to NLRP3 inflammasome-associated signalling (15, 16). Signal form, packaging, persistence, clearance, recipient-cell identity, and the surrounding metabolic state then determine whether these routes support type I interferon production and antigen presentation (18, 19) or instead sustain nuclear factor kappa B (NF-κB)-skewed inflammation (20, 21), programmed death-ligand 1 (PD-L1) expression (22), suppressive macrophage adaptation (20, 21, 23), and tissue injury (23). Redox remodelling is therefore not only an upstream source of mtDNA damage; it also changes the competence of tumour and immune cells to interpret mitochondrial material (24, 25).

We organise these divergent outcomes along an acute immune activation–chronic immune adaptation continuum. This is not a simple distinction between short and long exposure. A single intense treatment-associated release, repeated transient leakage, persistent low-level exposure, or inefficient clearance can follow different trajectories according to mtDNA oxidation and packaging, the route of delivery, the recipient compartment, and the pre-existing liver environment. Productive signalling requires temporal coordination between danger release, antigen availability, competent sensing, antigen presentation, effector recruitment, and resolution. When mitochondrial danger is recurrent or unresolved, it can instead be incorporated into suppressive myeloid differentiation, lymphocyte dysfunction, vascular and stromal restriction, and spatial immune escape.

**Figure 1:**
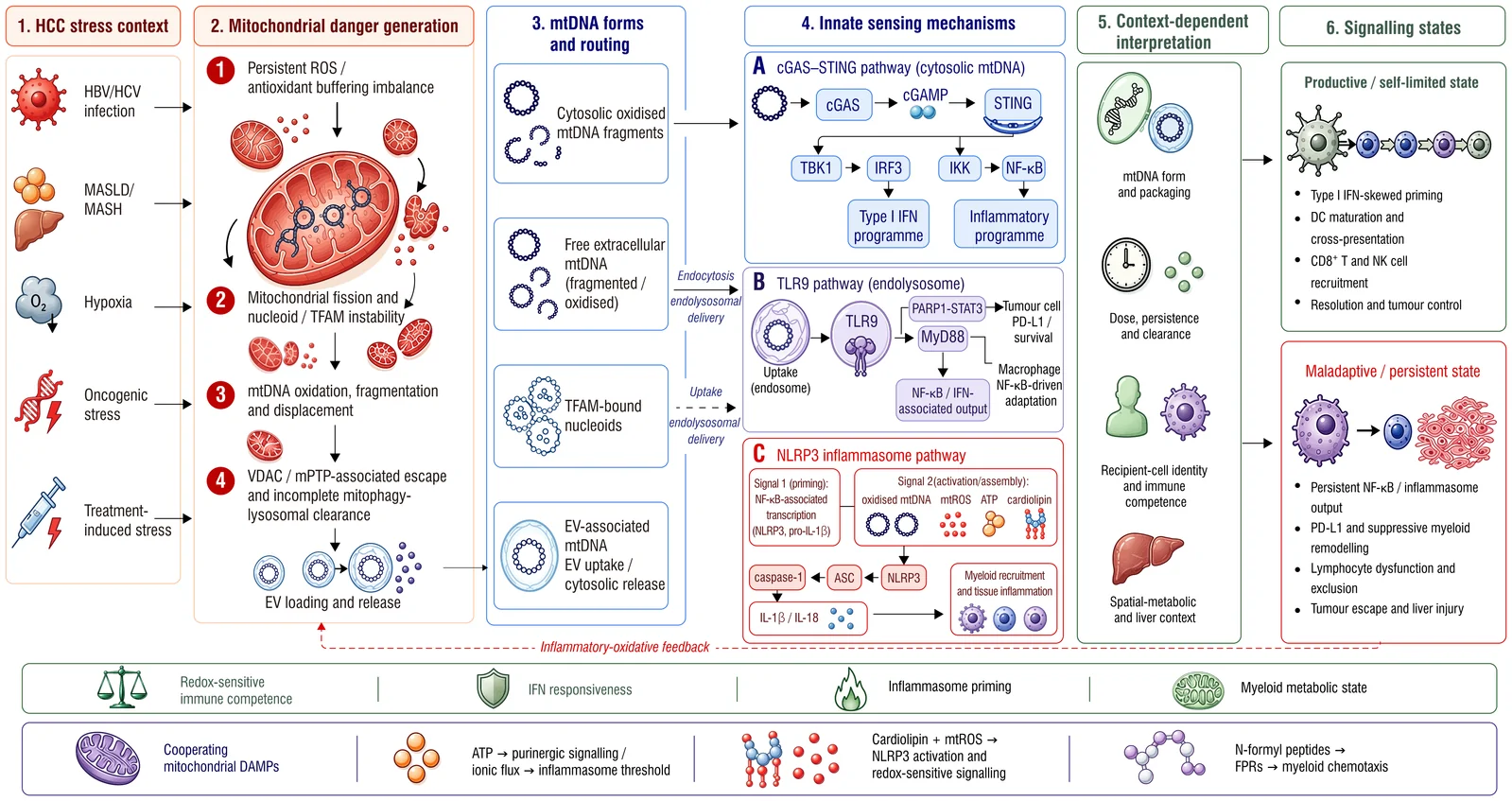
Redox-driven mitochondrial DNA danger signalling and context-dependent innate immune interpretation in hepatocellular carcinoma. HCC-associated stress promotes mitochondrial dysfunction, nucleoid instability and mtDNA mobilisation into cytosolic, free extracellular, protein-associated and extracellular-vesicle-associated forms. Representative routing–sensor relationships include cytosolic mtDNA–cGAS–STING and endolysosomal mtDNA–TLR9 signalling, whereas oxidised mtDNA contributes to NLRP3 activation together with other activation-associated mitochondrial inputs (13, 15–18, 20, 21, 23, 37, 38, 40, 41, 44, 45). For visual clarity, only representative routing–sensor relationships are drawn: EV-associated mtDNA can gain cytosolic access after uptake, whereas oxidised mtDNA is represented within NLRP3 Signal 2 rather than by an additional cross-panel arrow. Signal form and packaging, dose, persistence and clearance, recipient-cell competence, redox state and liver context determine whether mitochondrial danger supports a productive, self-limited immune state or maladaptive, persistent immune remodelling. Cooperating mitochondrial DAMPs modify signalling thresholds, and the dashed red feedback route denotes reinforcement of mitochondrial stress by chronic inflammatory and oxidative signalling.

mtDNA is the principal organising signal in this Review, but it is released within a broader mitochondrial danger environment. Extracellular ATP and related nucleotides alter purinergic signalling and inflammasome thresholds (26, 27); cardiolipin and mitochondrial reactive oxygen species (ROS) influence inflammasome and redox-sensitive pathways (15, 28); and N-formyl peptides regulate chemotaxis through formyl peptide receptors (29, 30). We treat these molecules as cooperating modifiers of mtDNA access and interpretation rather than as independent axes. The Review first examines how HCC metabolism and mitochondrial quality control generate structurally distinct mtDNA signals, then follows those signals through compartment-specific sensing and intercellular communication, before integrating them with immune-state transition and therapeutic timing. Direct HCC mechanistic evidence is distinguished from HCC-associated observations, adjacent mechanistic evidence, and the integrative model proposed here. **Figure 1** summarises the proposed redox–mtDNA framework, linking mitochondrial danger generation and mtDNA routing to compartment-specific innate sensing, context-dependent interpretation, and signalling states.

To develop this framework, we searched PubMed, Web of Science, and Google Scholar for English-language studies on HCC, redox signalling, mitochondrial stress, mtDNA release and sensing, intercellular communication, and immunotherapy; the search was updated in July 2026. The synthesis prioritises primary mechanistic studies in HCC, followed by HCC-associated evidence and, where direct data are unavailable, mechanistic studies from adjacent disease settings.

## 2 Upstream redox remodelling and mitochondrial DNA danger signalling in HCC

The mitochondrial stress relevant to HCC is rarely an abrupt collapse of organelle function. More often, malignant cells remain viable while compensating for a persistent burden of oxidative and metabolic injury. This partially compensated state permits membrane damage, nucleoid disorganisation, and oxidised mtDNA to accumulate without immediate cell death. The same redox programmes that preserve tumour-cell fitness also alter the responsiveness of neighbouring immune cells, linking signal generation to signal interpretation from the outset.

### 2.1 Metabolic plasticity and redox imbalance in HCC

HCC metabolism cannot be reduced to a binary switch from oxidative phosphorylation (OXPHOS) to glycolysis. Many tumours combine increased glucose uptake, glycolytic flux, lactate production, and extracellular acidification with retained mitochondrial respiration and tricarboxylic acid cycle activity (12, 13, 31). Substrate use changes across oxygen and nutrient gradients and can vary within the same lesion (31, 32). For mtDNA biology, the important point is that electron transport continues under fluctuating oxygen availability, oncogenic signalling, and antioxidant demand.

The coexistence of glycolytic adaptation and preserved mitochondrial activity creates a sustained but dynamically buffered redox burden. Electron transport, oncogenic signalling, and intermittent hypoxia increase mitochondrial ROS, whereas antioxidant programmes prevent oxidative stress from becoming immediately lethal (24, 28). Compensation preserves proliferation but does not necessarily restore organelle integrity. Because mtDNA lies close to the respiratory chain and depends on nucleoid packaging and mitochondrial repair, prolonged oxidative pressure can generate lesions and structural instability in cells that remain metabolically competent (15, 16).

Lactate, acidosis, and hypoxia also condition the cells that will later encounter mitochondrial material. These factors restrict T- and NK-cell activity, alter macrophage behaviour, promote vascular abnormality, and can increase PD-L1-associated immune restraint (33–36). Their relevance here is not that they require mtDNA sensing, but that they establish different thresholds for interpreting an mtDNA signal. A hypoxic macrophage or dendritic cell is not equivalent to the same cell in a well-perfused, metabolically permissive region.

### 2.2 Lipid and amino-acid metabolism as redox-buffering stress programmes

Lipid and amino-acid metabolism help sustain this compensated state. Lipogenesis, fatty acid oxidation (FAO), glutamine utilisation, and one-carbon metabolism can support membrane synthesis, ATP production, nicotinamide adenine dinucleotide phosphate regeneration, glutathione availability, and biosynthetic flexibility (12, 13). Continued FAO and OXPHOS in selected tumours therefore need not indicate metabolic normality; they may instead maintain viability while mitochondrial repair remains incomplete.

This adaptation creates a mechanistic paradox. Redox buffering protects tumour cells from acute oxidative death, yet it can prolong the survival of mitochondria that are structurally or functionally compromised. When antioxidant capacity prevents collapse but does not restore membranes, nucleoids, and quality control, oxidised proteins, lipids, and mtDNA persist. Repeated cycles of injury and compensation thus regulate not only tumour growth but also the quantity, molecular form, and timing of mitochondrial danger available for cytosolic displacement or extracellular release (13, 15).

### 2.3 Mitochondrial dynamics, quality control, and mtDNA mobilisation

Mitochondrial fusion, fission, and mitophagy normally preserve organelle integrity by redistributing contents, separating damaged regions, and removing mitochondria that cannot recover (13, 15). In HCC, dysregulation of mitofusin 1/2, optic atrophy 1, and dynamin-related protein 1 alters mitochondrial morphology and stress tolerance (13). Fission can support proliferation, but excessive fragmentation increases membrane stress and segregates damaged mitochondrial segments. When mitophagy–lysosomal clearance is incomplete, these segments remain sources of ROS and mtDNA leakage. HCC models directly connect fission-induced mtDNA stress with chemokine-dependent macrophage infiltration and tumour progression (37), establishing organelle dynamics as an upstream determinant of immune communication.

Nucleoid organisation determines whether persistent mitochondrial stress is converted into an accessible DNA signal. Transcription factor A, mitochondrial (TFAM), compacts and protects mtDNA while supporting replication and transcription (15, 38). Oxidative injury, altered dynamics, or reduced TFAM protection can loosen nucleoid architecture and facilitate mtDNA oxidation, fragmentation, and displacement. In HCC cells, TFAM downregulation increases cytosolic mtDNA and NLRP3-associated cytokine signalling, linking a tumour-cell-intrinsic defect in nucleoid maintenance to macrophage recruitment and tumour progression (23). This evidence supports TFAM-dependent mobilisation in HCC, but not the assumption that every oxidised or fragmented mtDNA species follows the same route.

Mechanistic studies outside HCC identify several non-exclusive routes by which displaced mtDNA crosses mitochondrial membranes, spanning sublethal leakage and cell-death-associated permeabilisation. During mitochondrial apoptosis, BAX/BAK-dependent outer-membrane permeabilisation can generate large macropores that permit inner-membrane herniation and mtDNA efflux (39). In oxidatively stressed non-apoptotic cells, oligomerised voltage-dependent anion channels (VDACs) form pores that release short mtDNA fragments (40). In activated macrophages, mitochondrial permeability transition pore (mPTP) opening can promote VDAC oligomerisation and the release of oxidised fragments that subsequently engage NLRP3 or cGAS–STING (41). Mitochondrial-derived vesicles and defective mitophagy provide additional routes in other experimental settings (15, 38). Together, these observations indicate that mtDNA mobilisation is not restricted to a single cellular state: viable stressed cells and dying cells can expose mitochondrial genomes through partly distinct mechanisms. Their relative contributions in HCC remain unresolved.

Once mtDNA reaches the cytosol or extracellular space, packaging becomes an upstream determinant of receptor access rather than a passive consequence of release. mtDNA may remain as cytosolic fragments, circulate freely, associate with TFAM or other proteins, or be protected within extracellular vesicles (15, 38). Free extracellular mtDNA is vulnerable to degradation and can reflect injury to tumour cells or non-malignant hepatocytes (15, 42). Protein association and vesicular encapsulation alter persistence, uptake, and recipient-cell selectivity. In HCC, hypoxic tumour cells transfer vesicle-associated mtDNA to macrophages (20), whereas treatment-associated injury can increase extracellular mtDNA available for macrophage endocytosis and TLR9 signalling (21). Signal architecture is therefore established before sensor selection: oxidation and fragmentation influence molecular reactivity, while packaging and trafficking determine which cellular compartment encounters the signal.

### 2.4 Redox signalling as a dual regulator of innate immune responses

ROS are signalling molecules as well as mediators of molecular damage. Changes in redox state alter cysteine-dependent kinases and phosphatases, receptor-proximal signalling, transcription-factor activation, and the metabolic programmes that support innate immune responses (24, 25, 28). Redox remodelling has two linked effects: it promotes mitochondrial and nucleoid injury, and it modifies the signalling thresholds of cells that generate or receive mtDNA. This dual action explains why mtDNA abundance alone is an incomplete predictor of immune outcome.

Recipient-cell competence is particularly important for interferon and NF-κB outputs. In plasmacytoid dendritic cells, mitochondria-derived ROS and metabolic checkpoints regulate TLR7/9-associated type I interferon responses (43). More broadly, redox-sensitive signalling influences STING- and TLR-associated kinase activity, interferon responsiveness, and NF-κB-dependent transcription (24, 25). A dendritic cell with intact interferon and antigen-processing programmes may translate nucleic-acid exposure into maturation and cross-presentation, whereas a macrophage conditioned by tumour-derived cytokines may favour persistent NF-κB activity and suppressive differentiation. In HCC, direct evidence centres on the divergent cellular programmes triggered after mtDNA delivery (18, 20–23); the individual redox-sensitive switches that bias these outcomes are less clearly mapped.

Redox state also sets inflammasome competence. NF-κB-dependent priming increases NLRP3 and pro-interleukin-1 beta (44), whereas mitochondrial ROS, ATP-associated ionic flux, oxidised mtDNA, cardiolipin, and defective organelle clearance can contribute to activation (15, 16, 27, 41, 45). Hypoxia, lactate, lipid accumulation, and previous inflammatory exposure also leave metabolic and epigenetic histories that alter later innate responses (34, 46). An ATP-rich, oxidised, hypoxic niche therefore contains not only more danger signals but recipient cells whose activation thresholds have already shifted.

DDX3 and biological sex provide narrower examples of this context dependence. In HCC, reduced DDX3 expression increases extracellular-vesicle release and alters vesicular cargo (47). Outside HCC, DDX3 has been linked to broader immune–metabolic regulation (48), DRP1-dependent mitochondrial fission and fatty-acid oxidation in metastatic breast cancer (49), and oxidative-stress-responsive activity in melanoma cells (50). DDX3 also contributes to sex-dependent variation in TLR-induced interferon-alpha production by plasmacytoid dendritic cells (51). These modifiers act on immune competence rather than constituting separate HCC mtDNA pathways, reinforcing the need to interpret cGAS–STING, TLR9, and NLRP3 in their recipient-cell context.

## 3 Mitochondrial DNA sensing and context-dependent innate immune signalling in HCC

mtDNA becomes immunologically active when it leaves the mitochondrial matrix and gains access to the cytosol, endolysosomal system, or extracellular environment (15, 38). Sequence alone does not determine immunogenicity. Oxidation, fragmentation, protein or vesicle packaging, concentration, persistence, and delivery to a particular cell govern receptor access and signal duration (15, 16). cGAS–STING, TLR9, and NLRP3-associated signalling are therefore better understood as compartment-specific interpretations of mitochondrial stress than as interchangeable responses to a uniform ligand. Parallel mitochondrial RNA leakage can also engage RIG-I/MDA5–MAVS signalling in other inflammatory settings, although the relevance of this pathway to HCC remains poorly defined (52).

### 3.1 Cytosolic mtDNA sensing through cGAS–STING

Cytosolic double-stranded mtDNA can bind cGAS and stimulate synthesis of the second messenger 2′3′-cyclic GMP–AMP (cGAMP) (17, 38). cGAMP activates endoplasmic-reticulum-resident STING and promotes its trafficking to the Golgi, where activation-associated palmitoylation supports signalling-competent STING (17, 38, 53). STING then recruits TANK-binding kinase 1 (TBK1) and supports interferon regulatory factor 3 (IRF3) phosphorylation; the TBK1–IRF3 branch promotes type I interferon transcription, whereas STING-associated IκB kinase (IKK)–NF-κB signalling can induce inflammatory cytokine, survival, and stress-adaptation programmes (17, 38). Although these trafficking and adaptor steps are established largely outside HCC, they provide the molecular scaffold through which malignant hepatocytes, macrophages, and antigen-presenting cells can generate distinct outputs.

The cellular recipient determines how this cascade is translated into immunity. In competent antigen-presenting cells, a coordinated and time-limited cGAS–STING response can support dendritic-cell maturation, cross-presentation, and CD8+ T-cell priming (19). In macrophages, persistent or metabolically conditioned STING/NF-κB signalling can instead maintain inflammatory cytokines and suppressive myeloid adaptation. Hypoxic HCC cells provide direct evidence for the latter route: vesicle-associated mtDNA is transferred to macrophages and activates cGAS–STING/NF-κB signalling linked to immunosuppressive polarisation and tumour progression (20). Tumour-cell-intrinsic STING may likewise generate interferon or inflammatory outputs, but in HCC its net effect is likely to depend on whether immune visibility outweighs checkpoint and survival adaptation.

Treatment can create a temporary cGAS–STING opportunity when mitochondrial injury, tumour-antigen release, and competent immune sensing are aligned (18, 54). Cabozantinib induces mitochondrial depolarisation, oxidative stress, and cytosolic mtDNA release in HCC models; STING agonism further increases immune-cell infiltration and cytotoxic activity (18). More recent HCC models show that STING agonism can also be accompanied by adaptive resistance involving increased intratumoral B-cell infiltration and immunosuppressive B-cell responses (55). These findings suggest that a STING-responsive window may be transient, and its immunological value depends on the receiving cellular compartment and the surrounding immune state. Macrophage dominance, B-cell-mediated adaptation, chronic liver inflammation, defective antigen presentation, or prolonged NF-κB output could redirect the response towards maladaptive inflammation.

### 3.2 Endosomal mtDNA sensing through TLR9

Extracellular or vesicle-associated mtDNA can be internalised by endocytosis and delivered to acidified endolysosomal compartments, where TLR9 recognises CpG-rich DNA (16, 17). Ligand-engaged TLR9 recruits myeloid differentiation primary response 88 (MyD88), initiating kinase and transcriptional programmes that converge on NF-κB and interferon-regulatory-factor-associated output (17). Unlike cytosolic cGAS sensing, this route is governed by uptake, endosomal maturation, vesicle trafficking, receptor abundance, and ligand persistence.

The consequence of TLR9 engagement depends on the receiving cell. In malignant hepatocytes, cell-intrinsic TLR9 activation promotes PD-L1 transcription through PARP1–STAT3 regulation, supporting immune escape (22). This places an endosomal DNA sensor within a tumour-cell adaptation programme and shows that receptor engagement can reinforce immune escape rather than productive immunity.

A parallel HCC route operates in macrophages during treatment. Sorafenib-associated mtDNA is released from injured tumour cells, taken up by macrophages, and routed through TLR9–NF-κB signalling, promoting M2-like polarisation and treatment resistance (21). Free extracellular mtDNA is vulnerable to degradation (15), and circulating fragmentomic changes in HCC do not identify a recipient pathway (42). Vesicular or protein-associated material can persist and be delivered more selectively (20, 21). Here, uptake and intracellular routing—not release alone—convert treatment injury into a macrophage programme of resistance.

### 3.3 NLRP3 inflammasome-associated mitochondrial stress signalling

NLRP3 inflammasome activity is organised into priming and activation steps, not direct receptor–ligand recognition alone. Signal 1 is supplied by NF-κB-activating pattern-recognition or cytokine receptors, which increase NLRP3 and pro-interleukin-1 beta expression and license subsequent assembly (44). Signal 2 can arise from ATP-associated ionic flux, mitochondrial ROS, oxidised mtDNA, cardiolipin exposure, lysosomal stress, or insufficient clearance of damaged mitochondria (15, 16, 27, 41, 45). These inputs promote NLRP3 oligomerisation, recruitment of the adaptor apoptosis-associated speck-like protein containing a CARD (ASC), and caspase-1 activation, leading to maturation of interleukin-1 beta and interleukin-18 (15, 16). The two-step architecture explains why an equivalent mtDNA burden can produce different cytokine outputs in cells with different priming histories.

In HCC cells, TFAM downregulation establishes a direct connection between nucleoid instability and inflammasome-associated immune remodelling. Loss of TFAM increases cytosolic mtDNA, activates NLRP3, and raises interleukin-1 beta and interleukin-18 production (23). These cytokines promote macrophage recruitment, M2-like polarisation, and tumour progression, whereas depletion of cytosolic mtDNA or NLRP3 blockade attenuates the phenotype (23). This source–signal–sensor–effector chain shows how a tumour-cell-intrinsic nucleoid defect can reorganise the surrounding myeloid compartment without requiring inflammasome assembly in the recruited macrophage.

Oxidised mtDNA is one NLRP3-associated input, not a unique or universally sufficient ligand. General mechanistic studies show that oxidised fragments can support NLRP3 activation (41, 45), but the response usually emerges from convergence with priming, ionic flux, mitochondrial ROS, cardiolipin, and organelle-clearance failure. Transient inflammasome activation can participate in damage recognition, whereas persistent interleukin-1 beta and interleukin-18 release in fibrotic, steatotic, or chronically inflamed liver can sustain myeloid recruitment and tissue injury. HCC directly supports TFAM–mtDNA–NLRP3 coupling (23), while the contribution of ATP, cardiolipin, and other co-activating inputs within HCC remains to be resolved.

### 3.4 mtDNA within the broader mitochondrial danger landscape

Damaged mitochondria release a composite signal rather than pure mtDNA. Extracellular ATP and related nucleotides engage purinergic receptors and alter potassium and other ionic fluxes, changing inflammatory recruitment and inflammasome thresholds (26, 27). Cardiolipin exposure and mitochondrial ROS reinforce NLRP3 and other redox-sensitive signalling (15, 16, 28). N-formyl peptides activate formyl peptide receptors (FPRs), recruiting or positioning myeloid cells that may subsequently encounter mtDNA (29, 30).

These signals interact before and after receptor engagement. ATP-driven ionic flux can lower the threshold for NLRP3 activation, cardiolipin and oxidised mtDNA can converge on inflammasome-associated signalling, and ROS alter both ligand production and recipient-cell competence (15, 16, 27, 28). Vesicular packaging can co-deliver mtDNA with lipids or proteins that modify uptake and intracellular routing (20). The result is not a single mitochondrial-DAMP cascade but a set of cooperating signals that determine where, when, and how strongly mtDNA is sensed.

mtDNA remains the organising signal because it provides a traceable connection between mitochondrial injury and cGAS–STING, TLR9, and NLRP3-associated routes in cytosolic, extracellular, protein-associated, and vesicular forms. Cooperating mitochondrial DAMPs do not displace that role; they influence which cells encounter mtDNA and how strongly or persistently those cells respond.

### 3.5 Acute immune activation-chronic immune adaptation continuum

The opposing effects of mtDNA sensing can be organised along an acute immune activation–chronic immune adaptation continuum. At one pole, a spatially restricted burst of mitochondrial danger is followed by efficient sensing and clearance. Type I interferon production, dendritic-cell activation, antigen presentation, and effector recruitment can then occur within a window in which tumour antigens and innate signals are temporally coordinated (16, 18, 19). At the other pole, repeated release or persistent low-level leakage occurs in tissue already conditioned by hypoxia, fibrosis, metabolic dysfunction, or chronic viral inflammation, and the response is incorporated into a stable tumour-supporting programme.

The transition between these poles depends on more than duration. Recurrent mtDNA exposure can repeatedly recruit monocytes and macrophages, while sustained NF-κB and inflammasome-associated cytokines alter differentiation and reinforce local oxidative stress (21, 23, 37). Antigen-presenting cells exposed to persistent metabolic constraints may lose the capacity to support effective priming (24, 56). At the same time, hypoxia, lactate, nutrient competition, and checkpoint signalling reduce cytotoxic lymphocyte fitness (33, 57, 58) and favour regulatory persistence (59). Inflammation may remain abundant as tumour surveillance declines.

Signal form, clearance, and recipient competence also determine position on the continuum. A single high-intensity injury may cause widespread tissue damage and suppressive myeloid recruitment, whereas repeated transient releases can accumulate when clearance is inefficient. Oxidised, fragmented, freely circulating, protein-associated, and vesicle-associated mtDNA differ in stability and routing; tumour cells, macrophages, dendritic cells, and endothelial cells differ in receptor expression and redox competence. Opposing states can therefore coexist in different regions or appear sequentially during treatment.

The continuum is a functional model of coordinated resolution versus chronic adaptation, not an established clock or universal sequence. Productive signalling combines innate activation with antigen presentation, effector recruitment, tumour control, and subsequent resolution. Maladaptive signalling combines persistent mitochondrial danger with suppressive myeloid dominance, impaired cytotoxic function, spatial exclusion, and ongoing liver injury. This distinction allows cGAS–STING, TLR9, and NLRP3-associated outputs to be compared without assigning any pathway a fixed antitumour or protumour identity.

### 3.6 HCC-specific immunobiological context

The redox–mtDNA response is superimposed on the distinctive immunobiology of the liver (6, 7). Kupffer cells, recruited monocytes, liver sinusoidal endothelial cells, stellate cells, and hepatocytes maintain tolerance to food-derived and microbial antigens while retaining the capacity to respond to infection and injury (6, 7). Chronic HBV or HCV infection repeatedly engages nucleic-acid sensing and interferon programmes (7); alcohol-related disease increases gut-derived and oxidative stress (1); MASLD/MASH combines lipotoxicity, metabolic dysfunction, and inflammatory T-cell responses (5, 60); and cirrhosis creates a fibrotic, hypoxic, and vascularly abnormal environment (1, 6).

These backgrounds alter multiple stages of mitochondrial danger signalling. Baseline ROS production and quality control influence signal generation; extracellular-DNA degradation and phagocytic clearance influence persistence; myeloid differentiation and interferon competence influence interpretation; and fibrosis and vascular dysfunction influence access. MASH-associated HCC is instructive because dysfunctional or auto-aggressive T-cell responses can limit checkpoint benefit despite an inflammatory environment (60). Increasing innate signalling in such a liver could intensify tissue injury without restoring coordinated tumour surveillance.

Aetiology and hepatic reserve are therefore mechanistic variables rather than demographic annotations. The same treatment-induced mtDNA signal may be transient and immunogenic in a well-perfused lesion with competent antigen-presenting cells, yet persistent, macrophage-dominant, or hepatotoxic in a fibrotic liver. This conditioning becomes especially important when mitochondrial danger propagates between tumour, immune, stromal, and vascular compartments.

## 4 Intercellular communication driven by redox–mtDNA signalling in the HCC tumour immune microenvironment

The most informative evidence follows source–signal–route–recipient–outcome chains; isolated pathway activation is less informative. In HCC, the best-defined routes originate in malignant cells and terminate in malignant cells, macrophages, or antigen-presenting-cell programmes. These routes explain how mitochondrial injury becomes a multicellular immune state rather than a cell-autonomous stress response.

### 4.1 Tumour-myeloid communication and suppressive myeloid remodelling

Hypoxic HCC cells provide the clearest example of suppressive intercellular transfer. Hypoxia promotes extracellular-vesicle-mediated export of mtDNA, which is taken up by macrophages and activates cGAS–STING/NF-κB signalling, driving suppressive polarisation and tumour progression (20). This pathway differs from cell-autonomous cGAS–STING activation because the same class of ligand is generated in the tumour cell but interpreted by a macrophage. Signal transfer, not merely signal production, determines the tissue-level outcome.

Other HCC-supported routes also converge on myeloid remodelling. During sorafenib exposure, tumour-derived extracellular mtDNA is taken up by macrophages and activates TLR9–NF-κB signalling, promoting M2-like polarisation and resistance (21). Within tumour cells, TFAM downregulation destabilises nucleoids, increases cytosolic mtDNA, and activates NLRP3-associated interleukin-1 beta and interleukin-18 production, which recruits and polarises macrophages (23). Fission-induced mtDNA stress provides a related upstream programme that promotes chemokine-dependent macrophage infiltration (37).

These routes differ in packaging, sensing compartment, and effector molecule, but they share a central principle: the first competent recipient helps determine the tissue-level meaning of tumour mitochondrial stress. Capture by antigen-presenting cells within a limited interferon window can support priming, whereas macrophage uptake in a hypoxic or chronically inflamed niche can convert mitochondrial material into repair-like, angiogenic, or suppressive adaptation. Once established, macrophage-derived interleukin-10, transforming growth factor beta, angiogenic factors, and metabolites further weaken antigen presentation and cytotoxic immunity (61, 62), while local inflammation and oxidative stress feed back to mitochondrial injury.

### 4.2 Antigen presentation, cytotoxic fitness, and regulatory adaptation

The influence of redox–mtDNA signalling on lymphocytes is often indirect. When dendritic-cell maturation and antigen presentation coincide with tumour-cell injury, CD8+ T-cell priming can be enhanced (19). When macrophage-derived cytokines, inhibitory ligands, and metabolic competition dominate, antigen presentation becomes ineffective and recruited lymphocytes enter dysfunctional states (56, 62). HCC single-cell and spatial studies define the downstream landscape in which this transition unfolds—exhausted CD8+ T-cell programmes, suppressive myeloid populations, and regional immune exclusion—without assigning mtDNA a singular causal role (63–65).

Cytotoxic-cell mitochondrial fitness determines whether recruitment becomes durable tumour control. Continuous antigen stimulation, hypoxia, PD-1 signalling, and nutrient restriction suppress mitochondrial biogenesis and oxidative capacity in T cells (57, 58, 66). Lactate, low glucose, and adenosine impose additional restrictions on T- and NK-cell activity (33, 67). Persistent mtDNA-associated myeloid signalling can reinforce these constraints by consuming nutrients, sustaining suppressive cytokines, and worsening local hypoxia even when the initiating innate response was immunogenic.

Regulatory T cells are comparatively adapted to low-glucose, high-lactate, and lipid-rich conditions because they retain oxidative metabolism and resist selected metabolic stresses (59, 68, 69). Their enrichment need not result from direct mtDNA sensing. Suppressive myeloid signalling, poor antigen-presenting-cell function, and altered metabolite availability can favour regulatory persistence as cytotoxic populations lose fitness. NK- and NKT-cell surveillance is likewise conditioned by lactate, nutrient competition, bile-acid metabolism, and the hepatic niche (33, 70).

The downstream lymphoid phenotype is best understood as the gain or loss of coordinated immune function, not as a list of independent cell defects. Productive signalling requires antigen presentation, effector recruitment, metabolic fitness, and sustained cytotoxic activity. Maladaptive signalling combines defective priming, suppressive myeloid programmes, regulatory-cell persistence, and restricted tissue access. mtDNA influences this balance chiefly through the cells that process, present, or relay mitochondrial danger.

### 4.3 Stromal, vascular, and metabolic stabilisation of immune adaptation

Stromal and vascular architecture determine whether an mtDNA-triggered response resolves or becomes embedded within a persistent tumour-supporting programme. Fibrosis increases tissue stiffness and diffusion barriers, while disorganised vessels create hypoxia and uneven delivery of oxygen, nutrients, immune cells, and therapy (1, 6). These conditions increase mitochondrial stress in tumour cells, favour suppressive macrophage states, and reduce the likelihood that tumour antigens and innate signals reach competent antigen-presenting cells (13, 33, 62).

Once established, these niches can stabilise an initially reversible inflammatory response. Vascular dysfunction restricts effector-cell recruitment, stromal signals maintain myeloid and regulatory populations, and metabolic competition limits lymphocyte recovery even when antigen remains available. Vesicular transfer, cytokine exchange, and repeated cellular injury sustain mitochondrial danger within this environment, while hypoxia and inflammation reinforce the redox pressure that generated it. Spatial studies identify macrophage-rich, hypoxic, and metabolically distinct regions in HCC, including niches in which stem-like tumour populations, SPP1-positive macrophages, and poor immune access coexist (65, 71, 72). These observations support the presence of locally stabilised immune states, although they do not establish mtDNA as the initiating cause of every niche.

### 4.4 Spatial coexistence of productive and maladaptive communication states

**Figure 2:**
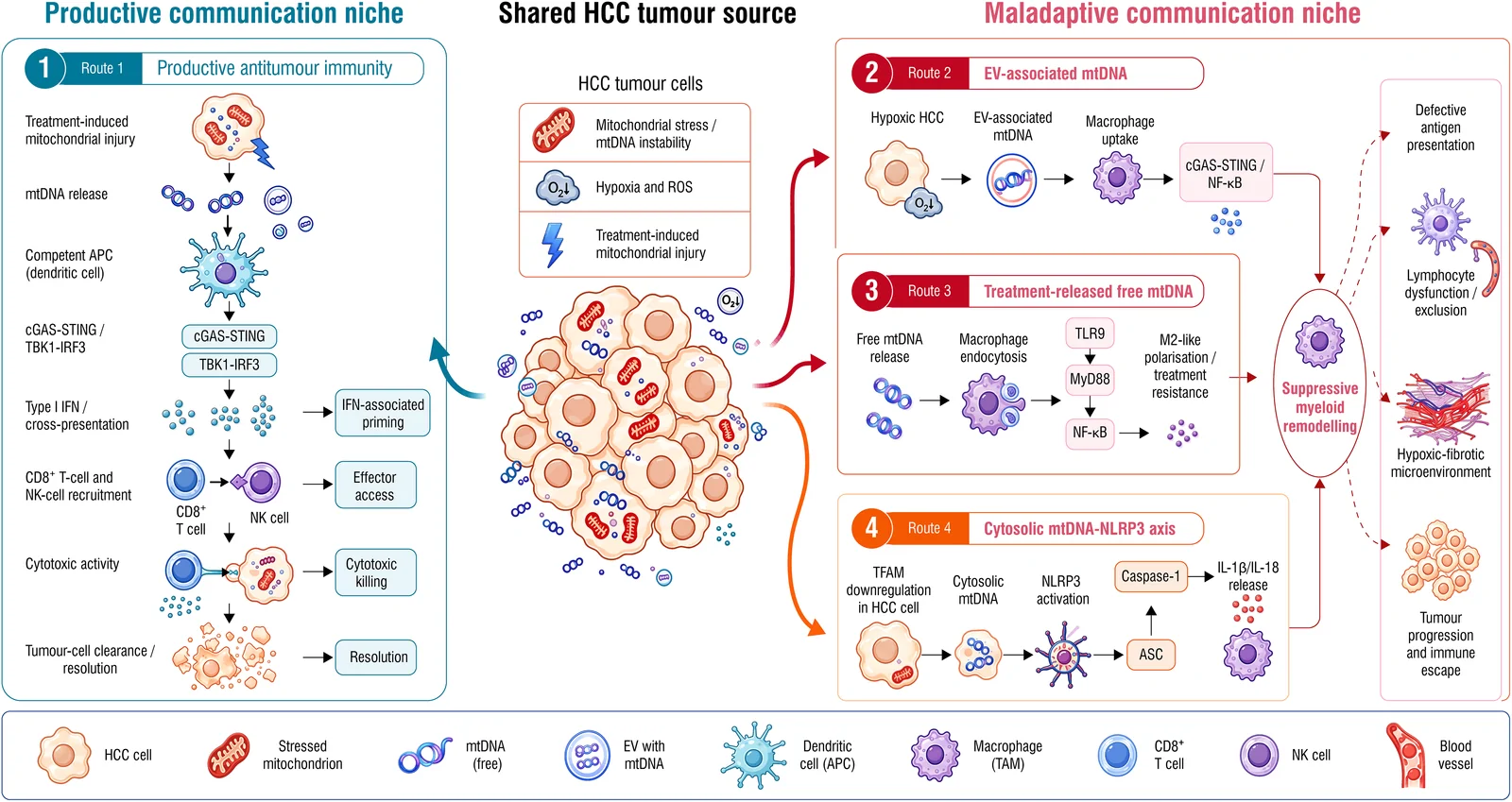
Spatially coexisting communication states within the hepatocellular carcinoma tumour immune microenvironment. Route 1 depicts a proposed productive communication state in which treatment-associated mitochondrial injury and mtDNA sensing are coordinated with competent antigen presentation, type I interferon signalling and cytotoxic-cell recruitment (18, 19). Routes 2–4 summarise representative HCC-supported maladaptive routes: hypoxic EV-associated mtDNA transfer to macrophages activates cGAS–STING/NF-κB signalling (20); treatment-released free mtDNA is endocytosed by macrophages and signals through TLR9–MyD88–NF-κB, promoting M2-like polarisation and treatment resistance (21); and TFAM downregulation in HCC cells drives cytosolic mtDNA–NLRP3–caspase-1–IL-1β/IL-18 signalling, which recruits and polarises macrophages (23). These routes may coexist spatially within the same lesion and converge on either tumour control or suppressive myeloid remodelling, lymphocyte dysfunction and immune escape.

Productive and maladaptive communication states need not occupy an HCC lesion uniformly. Regions in which mitochondrial danger is transient, efficiently cleared, and coordinated with antigen presentation may coexist with niches characterised by repeated mtDNA release, suppressive myeloid signalling, defective antigen presentation, and lymphocyte dysfunction. Transfer route, signal persistence, recipient-cell composition, vascular access, and local metabolic conditions determine the balance within each region. An interferon-responsive compartment may therefore lie adjacent to a hypoxic, macrophage-dominant, immune-excluded niche (65, 72). These states can also change during treatment, making the proposed continuum both spatial and temporal. **Figure 2** illustrates spatially coexisting productive and maladaptive communication states, including a proposed productive route and representative HCC-supported maladaptive mtDNA routes.

This heterogeneity complicates measurement. Bulk tissue profiles average biologically opposing states, whereas a single biopsy may sample only one compartment. Circulating mtDNA cannot distinguish tumour burden, non-malignant hepatocyte injury, treatment-associated damage, or impaired clearance (15, 42), and whole-tissue pathway activation does not reveal whether the dominant response originates in malignant hepatocytes, macrophages, dendritic cells, or stromal populations. Spatially resolved and longitudinal sampling is therefore required to connect a defined mitochondrial signal with its recipient cell, sensing pathway, and functional immune consequence. This dual organisation helps explain why circulating mtDNA abundance, whole-tissue pathway activation, or immune-cell density may fail to predict effective antitumour immunity.

## 5 From mitochondrial danger to immune adaptation: an integrated model in HCC

The value of the redox–mtDNA axis lies in treating it as a dynamic model, not a receptor list. It connects three levels of biology: generation and mobilisation of mitochondrial danger, the competence of recipient cells to interpret that danger, and consolidation of a multicellular immune state. No single receptor or cell type controls the entire process. cGAS–STING, TLR9, and NLRP3-associated routes sample different forms and compartments of mitochondrial injury, while redox, metabolic, spatial, and liver-disease conditions determine which output becomes dominant.

### 5.1 Signal generation and recipient-cell interpretation

The model begins with a stressed but viable tumour cell. Metabolic plasticity and antioxidant buffering sustain proliferation (13), while fission (37), nucleoid instability (23), incomplete mitophagy, hypoxia, and treatment injury increase mtDNA oxidation and displacement (15, 18, 38). Signal architecture is established before receptor selection: cytosolic fragments favour cGAS access; extracellular or vesicular material can enter endolysosomes and engage TLR9; and oxidised mtDNA can cooperate with ROS, ATP, cardiolipin, and ionic flux in NLRP3 activation (15, 16, 27, 41).

What happens next depends on recipient-cell competence. Antigen-presenting cells with intact interferon and antigen-processing programmes may translate mtDNA into maturation and cross-presentation (19). Macrophages conditioned by hypoxia, lactate, prior inflammation, or tumour-derived signals can favour NF-κB-skewed or M2-like responses (20, 21, 23), whereas malignant hepatocytes can use TLR9-associated signalling to increase PD-L1 (22). Other HCC stress programmes further regulate PD-L1 expression (73). Redox history and metabolic state (24, 43), DDX3-associated regulation (48, 49), and host factors such as biological sex (51) can modify signalling thresholds. Immune output therefore emerges from the interaction between signal architecture and cellular state.

### 5.2 Transition towards chronic immune adaptation

A productive response requires temporal coordination between mitochondrial injury, antigen availability, innate sensing, antigen presentation, effector recruitment, and resolution. When these steps are spatially separated or temporally mismatched, danger signalling can persist without effective tumour control. Repeated monocyte recruitment, macrophage polarisation (21, 23, 37), inflammasome-associated cytokines (23), and tumour-cell checkpoint adaptation (22) can then convert an initially reversible response into a self-reinforcing network.

Metabolic and spatial constraints consolidate this transition. Hypoxia and lactate reduce cytotoxic-cell fitness (33, 57, 58); abnormal vessels limit access and fibrosis restricts movement (1, 6); and regulatory populations persist under conditions that exhaust effector T cells (59, 68). Myeloid and stromal cells further amplify oxidative stress and suppressive cytokines. Mitochondrial danger then ceases to behave as a discrete alarm and becomes part of a tumour-supporting programme in which inflammation, repair, angiogenesis, and immune tolerance are coupled.

This model explains why an inflamed tumour may remain immunologically ineffective. Persistent interferon- or NF-κB-associated transcription does not guarantee competent antigen presentation, and abundant immune cells do not guarantee cytotoxic function. The meaningful distinction is between coordinated immunity that culminates in tumour control and chronic inflammatory adaptation that preserves the tumour while damaging the surrounding liver.

### 5.3 Biological tensions within the model

The first tension is whether mtDNA acts as a causal driver or as a marker of mitochondrial and hepatic injury. HCC studies involving extracellular-vesicle transfer, macrophage TLR9 sensing, tumour-cell NLRP3 activation, and treatment-induced STING signalling show that defined mtDNA forms can be functionally active (18, 20, 21, 23). Circulating mtDNA may nevertheless report tumour burden, hepatocyte injury, treatment damage, or impaired clearance without identifying the causal recipient pathway (15, 42). Driver status is strongest when a traceable source and defined molecular form are linked to delivery, recipient-pathway perturbation, and a relevant functional outcome.

A second tension is whether cGAS–STING, TLR9, and NLRP3 form a unified sequence or a set of parallel mechanisms. They can arise from related mitochondrial stress and converge on cytokine production, recruitment, and myeloid remodelling, but they need not occur in the same cell or in a fixed order. Vesicular mtDNA may activate macrophage cGAS–STING in one region, treatment-released material may engage TLR9 elsewhere, and tumour-cell nucleoid instability may activate NLRP3 in another compartment. The integrated axis is therefore a network of linked routes, not a single biochemical cascade.

A third tension concerns temporal transition and spatial coexistence. Productive and maladaptive responses can occupy different tumour regions and can change during treatment. This coexistence explains why a single biopsy or circulating marker may fail to predict response, but it also exposes the principal evidence gap: human thresholds for signal dose, persistence, clearance, and state transition remain undefined. The framework gains value by directing attention to how mitochondrial danger moves through time, space, and cellular compartments. **Table 1** summarises the evidence tiers and interpretive limits of this model.

**Table 1:** Evidence tiers and interpretive limits of the redox–mtDNA–innate immune framework in hepatocellular carcinoma.

Evidence tier	Representative evidence	Interpretation and principal uncertainty
Direct HCC mechanistic evidence	Tumour-cell TLR9–PD-L1 signalling; treatment-induced mtDNA–STING activation; hypoxic EV-mediated mtDNA transfer to macrophages; treatment-released mtDNA–TLR9–NF-κB signalling in macrophages; TFAM downregulation–cytosolic mtDNA–NLRP3 signalling; and fission-associated mtDNA stress with macrophage recruitment (18, 20–23, 37).	Defined source–signal–sensor–recipient links are experimentally supported in HCC. The complete sequence from redox remodelling to multicellular immune-state transition and therapeutic outcome has not been established.
HCC-associated evidence	Metabolic and redox heterogeneity; mitochondrial dysfunction; circulating mtDNA fragmentomic alterations; exhausted CD8+ T-cell states; suppressive myeloid and regulatory-cell programmes; and spatially organised immunometabolic niches (12, 13, 31, 32, 42, 62–65, 71, 72).	These findings establish disease relevance and immune-state association, but mtDNA-dependent causation is often unproven.
Adjacent mechanistic evidence	Mechanisms of mtDNA release and compartment-specific sensing; redox regulation of Toll-like receptor and interferon responses; inflammasome priming and assembly; innate immune memory; ATP–purinergic, N-formyl peptide–FPR, cardiolipin–NLRP3, and mitochondrial ROS signalling (15–17, 24–30, 38, 40, 41, 43–46, 48–51, 53).	These studies establish biological plausibility, but their contribution, cellular compartment, aetiological dependence, and host-modifier effects in HCC remain uncertain.
Integrative synthesis proposed in this Review	An acute immune activation–chronic immune adaptation continuum governed by mtDNA form, signal persistence and clearance, recipient-cell competence, spatial organisation, and background liver disease (15–18, 20–25, 38, 54, 60, 65, 72).	This is a testable synthesis rather than an established linear pathway. Human thresholds, causal state transitions, predictive biomarkers, and therapeutic windows remain undefined.

Evidence tiers reflect proximity to direct HCC mechanistic evidence, not formal quality grading.

## 6 Therapeutic implications of redox–mtDNA signalling in HCC

Neither activation nor inhibition of mitochondrial danger signalling has a fixed therapeutic meaning in HCC. Increasing mtDNA exposure may enhance antigen presentation when the signal reaches competent antigen-presenting cells within a limited window (18, 19). The same intervention can promote macrophage TLR9 signalling (21), tumour-cell NLRP3-associated cytokines (23), chronic NF-κB output (20, 21), or liver injury (16, 60). Therapeutic reasoning begins with the immune state and limiting function, not with an assumption that a sensor is inherently beneficial or harmful.

### 6.1 The therapeutic paradox of mitochondrial danger signalling

The central paradox is that tumour-cell injury is required for antigen release but can simultaneously generate suppressive mitochondrial signals. Cabozantinib-induced mitochondrial depolarisation and mtDNA release can be redirected through STING agonism to increase immune infiltration in HCC models (18). In contrast, sorafenib-associated mtDNA release promotes macrophage TLR9–NF-κB signalling, M2-like polarisation, and resistance (21). Hypoxic vesicular transfer and TFAM-associated NLRP3 activation organise additional tumour-supporting myeloid routes (20, 23). The therapeutic consequence of damage is set by where the material travels and which cell interprets it.

This paradox argues against simply maximising inflammation. Excessive or persistent activation could injure non-malignant hepatocytes, amplify fibrosis-associated inflammation, and reduce hepatic reserve, whereas indiscriminate suppression could remove signals required for antigen presentation and treatment-induced priming. The therapeutic objective is to create or preserve a productive window while preventing mitochondrial danger from being absorbed into chronic myeloid and tumour-cell adaptation.

### 6.2 Poorly primed tumours: creating a productive immune window

Poorly primed tumours may require coordinated generation of tumour antigens and innate signals; nonspecific pathway activation is unlikely to be sufficient. Treatment-induced mitochondrial injury can provide cytosolic mtDNA and antigenic material, while cGAS–STING support can promote type I interferon production and dendritic-cell cross-presentation when competent antigen-presenting cells are present (18, 19). Its benefit depends on directing the resulting inflammation into competent priming and is likely to be lost when antigen presentation remains defective or suppressive macrophages capture most of the released material.

Signal generation cannot be separated from packaging and clearance. Transient disruption of mitochondrial integrity may enhance immunogenicity when paired with competent sensing (18), whereas restoration of mitophagy–lysosomal clearance could reduce chronic leakage and inflammasome-associated inflammation (15, 38). In hypoxic tumours, interfering with suppressive vesicular transfer may be more selective than globally reducing mtDNA release because extracellular vesicles direct mitochondrial material towards macrophages (20). The therapeutic logic therefore rests on selective, time-limited control of signal routing rather than global manipulation of mtDNA release.

A coherent therapeutic sequence would use treatment-induced injury to establish priming before checkpoint blockade sustains the emerging tumour-reactive response. Current HCC trials demonstrate the activity of checkpoint-based therapy (8, 9, 74) but do not resolve whether such temporal alignment improves benefit. The relevant biological signature would be a transient rise in mtDNA signals and interferon activity, recovery of dendritic-cell competence, and subsequent cytotoxic-cell recruitment, followed by signal clearance rather than continued debris accumulation and macrophage uptake.

### 6.3 Macrophage-dominant maladaptive inflammation

In macrophage-dominant tumours, the receiving compartment may offer a more tractable target than total mtDNA abundance. HCC evidence identifies suppressive routes through hypoxic EV-mtDNA–cGAS–STING/NF-κB signalling (20), treatment-released mtDNA–TLR9–NF-κB signalling (21), and tumour-cell TFAM–mtDNA–NLRP3 cytokine production (23). Introducing additional mitochondrial injury into this state could strengthen rather than reverse immune suppression.

In this setting, the rational point of intervention may be TLR9–NF-κB output, NLRP3-associated cytokines, extracellular-vesicle uptake, or downstream macrophage differentiation, depending on where the dominant suppressive route resides. The central translational problem is therefore to distinguish tumour-cell, macrophage, and extracellular-transfer dominance rather than applying a common mtDNA-directed strategy across tumours.

The relevant endpoint is correction of the limiting immune function. Reduced pathway phosphorylation or cytokine abundance is insufficient if antigen presentation remains defective, macrophages retain suppressive programmes, or effector cells still fail to enter the tumour. A meaningful response is therefore reflected in myeloid state, antigen-presenting-cell competence, cytotoxic activity, and durable tumour control, not receptor engagement alone.

### 6.4 Hypoxic and immune-excluded tumours

Hypoxic and immune-excluded tumours present a different constraint: an innate signal may be generated but remain inaccessible to competent effector and antigen-presenting cells. Abnormal vessels, stromal restriction, lactate accumulation, and macrophage-dominant niches can redirect mtDNA towards local suppressive recipients while limiting cytotoxic-cell trafficking (6, 33, 35, 56). Increasing mitochondrial injury or STING activity alone may then intensify local inflammation without overcoming spatial exclusion.

When access is the dominant bottleneck, vascular or metabolic normalisation is biologically upstream of additional innate stimulation. Improved oxygenation, immune-cell entry, and antigen-presenting-cell function can create a compartment in which new danger signals are more likely to be interpreted productively, while limiting suppressive vesicular transfer or macrophage capture can redirect the recipient cell. In this setting, spatial readouts are more informative than bulk measures of pathway activation.

The preferred sequence follows the bottleneck: vascular and stromal correction before priming when access is limiting; myeloid reprogramming alongside tumour injury when macrophage capture dominates; and checkpoint blockade after antigen presentation has recovered. Because different regions of the same tumour can move towards productive and maladaptive states simultaneously, treatment effects are best interpreted longitudinally and in space.

### 6.5 Inflamed or hepatotoxic liver: biomarkers, timing, and safety

An inflamed or hepatotoxic liver requires a safety-first interpretation. Circulating cell-free mtDNA, oxidised mtDNA, extracellular-vesicle-associated mtDNA, and fragmentomic features provide complementary information about tumour and liver injury (15, 20, 42), but none identifies the recipient cell or distinguishes productive immunity from off-tumour inflammation. Their meaning emerges only when tissue and blood measurements are considered alongside pathway localisation, myeloid and antigen-presenting-cell phenotypes, cytotoxic-cell fitness, and conventional indicators of hepatic injury and reserve.

Spatial and temporal information is particularly valuable because beneficial and harmful responses may coexist. Biomarker interpretation is strongest when mitochondrial stress and mtDNA form can be linked to the cellular compartment of sensing, antigen presentation, macrophage state, effector-cell access, and subsequent resolution or persistence. The useful endpoint is not an exhaustive panel for every patient, but identification of the dominant route and the immune function that limits tumour control.

Liver disease context must remain part of therapeutic selection. HBV-, HCV-, alcohol-, and MASLD/MASH-associated HCCs arise in different inflammatory and metabolic environments, and cirrhosis limits tolerance for additional innate immune injury (1, 5, 7, 60). In mice with NAFLD, metformin restored CD8+ T-cell responses to immune checkpoint therapy (75), illustrating how host metabolism can condition treatment competence rather than simply add background comorbidity. Biological sex may modify selected interferon responses (51), while hepatic reserve determines whether antitumour inflammation can be separated from unacceptable tissue damage. An intervention that is rational in a non-cirrhotic model may therefore be unsuitable in active hepatitis, advanced fibrosis, or decompensated liver disease.

The practical value of the redox–mtDNA framework is to match intervention to state: create a priming window in poorly primed tumours, reprogramme suppressive recipients in macrophage-dominant disease, restore access in hypoxic immune-excluded lesions, and limit persistent leakage or off-tumour activation in inflamed or hepatotoxic liver. These are mechanistic working states rather than validated clinical strata. **Table 2** summarises the evidence needed to advance redox–mtDNA-directed strategies and the findings that would argue against further development.

**Table 2:** Minimum evidence and key limitations for translational development of redox–mtDNA-directed strategies in hepatocellular carcinoma.

Translational domain	Minimum evidence required	Findings that would limit further development
Causal mechanism	Definition of the mtDNA form, cellular source, delivery route, recipient compartment, and sensing pathway; causal perturbation and, where feasible, rescue experiments in HCC-relevant models (18, 20–23, 37).	Correlation without causality; dominant off-axis effects; or failure to reproduce the intended immune consequence after pathway perturbation.
Cellular and spatial context	Compartment-resolved identification of signal-generating and signal-receiving cells; spatial assessment of pathway activity, antigen presentation, myeloid state, lymphocyte fitness, and vascular access (20, 21, 23, 37, 63–65, 71, 72).	Opposing effects across cellular compartments; or bulk measurements that conceal spatially divergent immune states.
Temporal immune-state transition	Distinction between transient, recurrent, and persistent signalling; assessment of dose, sequence, clearance, recovery, and durable tumour control (15–18, 20, 21, 23–25).	Chronic inflammatory adaptation, immune tolerance, repeated tissue injury, or pathway activation without durable antitumour activity.
Pharmacodynamic and functional confirmation	Paired tissue–blood and longitudinal assessment; measurement of mtDNA abundance, oxidation, fragmentation, localisation, and vesicular packaging; and confirmation of sensing activity together with correction of the limiting immune function, including antigen-presenting-cell competence, myeloid state, lymphocyte fitness, or effector-cell access (15, 17, 20, 21, 42).	Nonspecific circulating mtDNA changes; receptor or cytokine activation without correction of the limiting immune function; or pathway engagement without durable tumour control.
Liver-specific safety and host context	Evaluation of HCC aetiology, cirrhosis, hepatic inflammation, functional reserve, off-tumour innate activation, and relevant host modifiers, including biological sex where mechanistically justified (1, 5–7, 51, 60, 75).	Worsening hepatitis, fibrosis, or hepatic decompensation; loss of functional reserve; or unacceptable systemic or off-tumour immune activation.

These are proposed preclinical and translational criteria, not validated clinical rules.

## 7 Conclusions

Direct HCC studies now support several source–signal–recipient routes. Treatment-induced mitochondrial injury can release mtDNA that contributes to STING-dependent immune remodelling (18); hypoxic HCC cells can transfer vesicle-associated mtDNA to macrophages and activate cGAS–STING/NF-κB signalling (20); treatment-released extracellular mtDNA can be routed through macrophage TLR9–NF-κB (21); and TFAM loss can connect nucleoid instability with tumour-cell NLRP3 activation and macrophage recruitment (23). These mechanisms establish that mtDNA can act as a functional signal in HCC, although they do not yet define a universal sequence operating across tumours.

The available evidence is best integrated by recognising that mtDNA abundance and receptor activation are not sufficient descriptors of immune outcome. Metabolic and antioxidant adaptation permit mitochondrial and nucleoid injury to accumulate; dynamics, incomplete clearance, membrane permeability, and treatment injury then mobilise mtDNA into cytosolic, free extracellular, protein-associated, or vesicle-associated forms. Signal architecture determines receptor access, while recipient-cell competence, persistence and clearance, spatial organisation, and chronic liver disease determine whether sensing supports productive immunity or chronic adaptation. ATP, cardiolipin, mitochondrial ROS, and N-formyl peptides modify access and signalling thresholds without replacing mtDNA as the organising signal.

The decisive next step is to establish in human HCC which mtDNA species arises from which cellular source, reaches which recipient compartment, and changes which immune function. A causal role cannot be inferred from circulating abundance alone; it becomes persuasive when localisation, pathway perturbation, and restoration or loss of tumour control converge. Spatially resolved longitudinal sampling is particularly important because tumour-derived signals and background liver injury can overlap while productive and maladaptive states evolve side by side. The translational implication is straightforward: intervention should follow the dominant biological bottleneck in its cellular and temporal context rather than activate or inhibit an innate sensor in isolation.

## Glossary

ATP: adenosine triphosphate
ASC: apoptosis-associated speck-like protein containing a CARD
cGAS: cyclic GMP–AMP synthase
cGAMP: 2′3′-cyclic GMP–AMP
cGAS–STING: cyclic GMP–AMP synthase–stimulator of interferon genes
DAMP: danger-associated molecular pattern
DC: dendritic cell
DDX3: DEAD-box RNA helicase 3
DRP1: dynamin-related protein 1
ETC: electron transport chain
ER: endoplasmic reticulum
EV: extracellular vesicle
FAO: fatty acid oxidation
FPR: formyl peptide receptor
HBV: hepatitis B virus
HCC: hepatocellular carcinoma
HCV: hepatitis C virus
ICI: immune checkpoint inhibitor
IKK: IκB kinase
IFN: interferon
IL-1β: interleukin-1 beta
IL-10: interleukin-10
IL-18: interleukin-18
IRF3: interferon regulatory factor 3
MASH: metabolic dysfunction-associated steatohepatitis
MASLD: metabolic dysfunction-associated steatotic liver disease
MFN1/2: mitofusin 1/2
mtDNA: mitochondrial DNA
mPTP: mitochondrial permeability transition pore
MyD88: myeloid differentiation primary response 88
NADPH: nicotinamide adenine dinucleotide phosphate
NF-κB: nuclear factor kappa B
NLRP3: NOD-like receptor family pyrin domain containing 3
NK cell: natural killer cell
NKT cell: natural killer T cell
OPA1: optic atrophy 1
OXPHOS: oxidative phosphorylation
PD-1: programmed cell death protein 1
PD-L1: programmed death-ligand 1
PINK1: PTEN-induced kinase 1
ROS: reactive oxygen species
STING: stimulator of interferon genes
TAM: tumour-associated macrophage
TBK1: TANK-binding kinase 1
TCA cycle: tricarboxylic acid cycle
TGF-β: transforming growth factor-β
TIME: tumour immune microenvironment
TLR9: Toll-like receptor 9
Treg: regulatory T cell
VDAC: voltage-dependent anion channel
